# Supplementary material for: Spatial transcriptomic analysis of Sonic hedgehog medulloblastoma identifies that the loss of heterogeneity and promotion of differentiation underlies the response to CDK4/6 inhibition
Source: Genome Med. 2023 May 1;15:29. doi: 10.1186/s13073-023-01185-4 (PMC10150495; doi:10.1186/s13073-023-01185-4)
Supplement: Supplementary file 1 — Additional file 1: Supplementary Figure S1AQ. Optimisation of the Spatial Transcriptomics technology for hybrid human-mouse tissue sections. This tiff file represents a graphical overview of the Visium Spatial Transcriptomics technology and the optimisation steps required for hybrid human-mouse tissue. Supplementary Figure S2. Structures of mouse histology can be identified within each sample using Visium spatial transcriptomics data, with high resolution and additional information about heterogeneity in gene expression present within one cluster. This tiff file represents a visual overview demonstrating how clustering analysis using gene expression data can define anatomically correct structures within the mouse cerebellum. Supplementary Figure S3. Spatial visualisation and quality control assessment of gene expression within Medulloblastoma Patient-derived orthotopic xenografts. This tiff file illustrates the total number of unique molecular identifiers, human genes and mouse genes per spot across the intact tissue section. Supplementary Figure S4. Diagnostic plots to assess technical variation and normalisation for gene expression analysis of Visium ST-seq data. This tiff file represents the normalisation and quality assessment of Visium ST-seq data across mouse, human and mixcompartments to identify differentially expressed genes in each compartment following Palbociclib treatment. The total number of unique molecular identifiers, human genes and mouse genes per spot across the intact tissue section. Supplementary Figure S5. Data-driven cell type detection for spatial transcriptomics spots were highly correlated to independent pathologist’s annotation based on histology. This tiff file illustrates independent pathologist annotationof Palbociclib A, B and control C overlaid with annotate cell types on the basis of Visium spot gene expression. Supplementary Figure S6. Overview of the reference single cell RNA-sequencingdata used for automated spot cell type identi [file 13073_2023_1185_MOESM1_ESM.pdf]

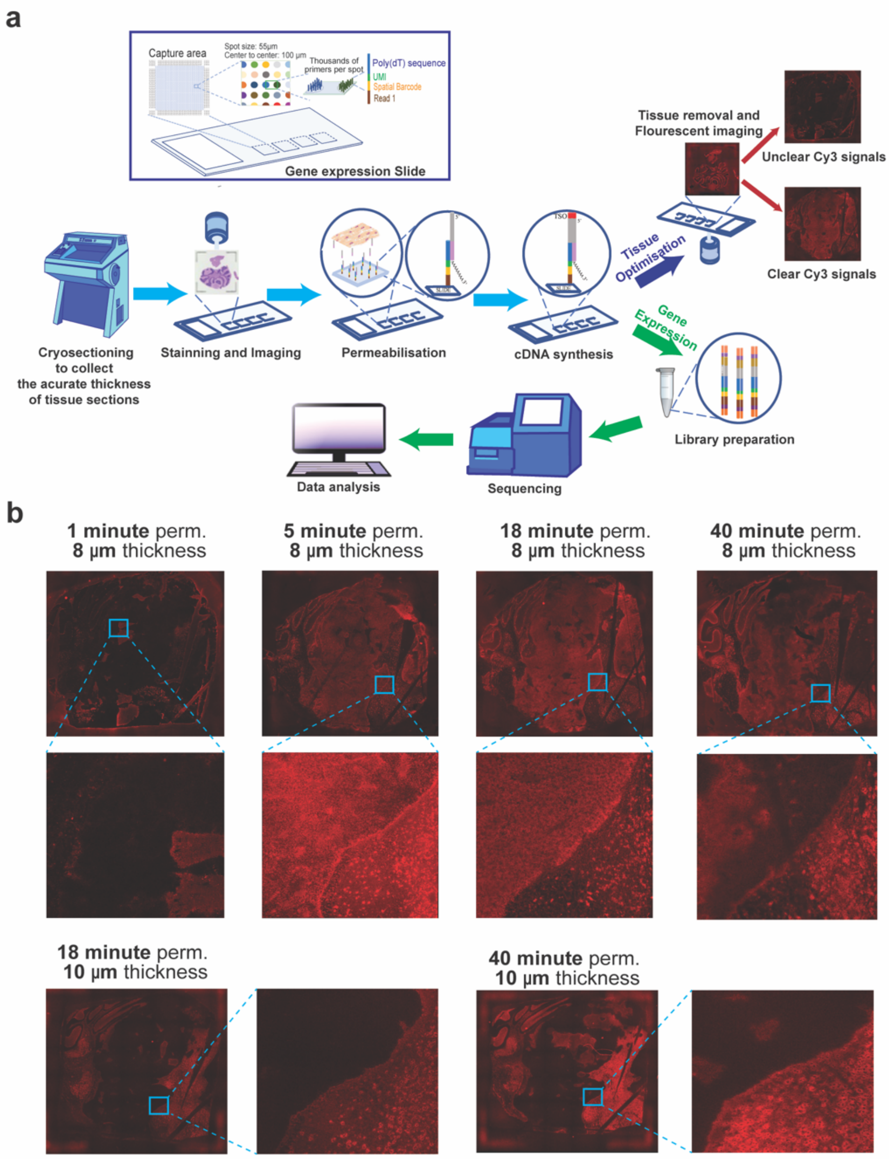

Supplementary Figure S1. Optimisation of the spatial Transcriptomics (Visium) technology for hybrid human-mouse tissue sections.

a. Visium array (described in small box). Top panel shows the design of an array-printed glass slide (Visium, 10x Genomics). Four capture areas (size of 6.5mm x 6.5 mm) are shown, each contains ~5,000 unique spatially barcoded spots. Spot size is 55μm with 100 μm centre to centre distance. Each spot has millions of probes with poly(dT) sequences, UMI, spatial barcode sequence, and Illumina Truseq read 1 adapter .

Visium workflow. Bottom panel shows visium workflow. Prior to library preparation (gene expression assay – GEx assay), Tissue Optimisation (TO assay) was performed to optimise permeabilisation conditions that worked for both human and mouse cells. In the TO step, tissue samples are sectioned, placed onto the microarray slide, fixed, stained, permeabilised. The released RNA is used for cDNA synthesis with fluorescence labelled nucleotides and the slide is imaged by microscopy to check for the mRNA capture efficiency. Based on the quality of the fluorescence signal across the whole sections, we found the optimal sectioning thickness and permeabilization time for the mouse PDOX system. In the actual gene expression GEx process, the library preparation is prepared after cDNA synthesis, followed by sequencing and data analysis. The cDNA is synthesised from the captured RNA molecules directly on the slide, and then released from the microarray and collected. The cDNA later is used to generate a sequencing library. Sequencing reads contain spatial information based on the unique spatial barcodes. Consequently, the gene expression measurements can be visualised and analysed together with their originated locations within the tissue and can be mapped to the high-resolution tissue image.

b. Tissue optimisation of MB-PDX. Distribution of Cy3-labelled cDNA signals (strong red suggests high signal; dark means low/no signal) across the human and mouse tissue sections were. For tissues sectioned at 10μm, across a range of permeabilisation time-points from 1 minute to 40 minutes, we consistently found that large areas, especially the tumour in the centre, showed no cDNA signal (dark regions). For tissues sectioned at 8μm we tested permeabilisation time-points ranged from 1 minute to 40 minutes. Except for the 1-minute permeabilisation option, other time-points showed cDNA signals across the entire tissue; however, the signal at 40 minute permeabilisation was highly diffused, suggesting over-permeabilization.

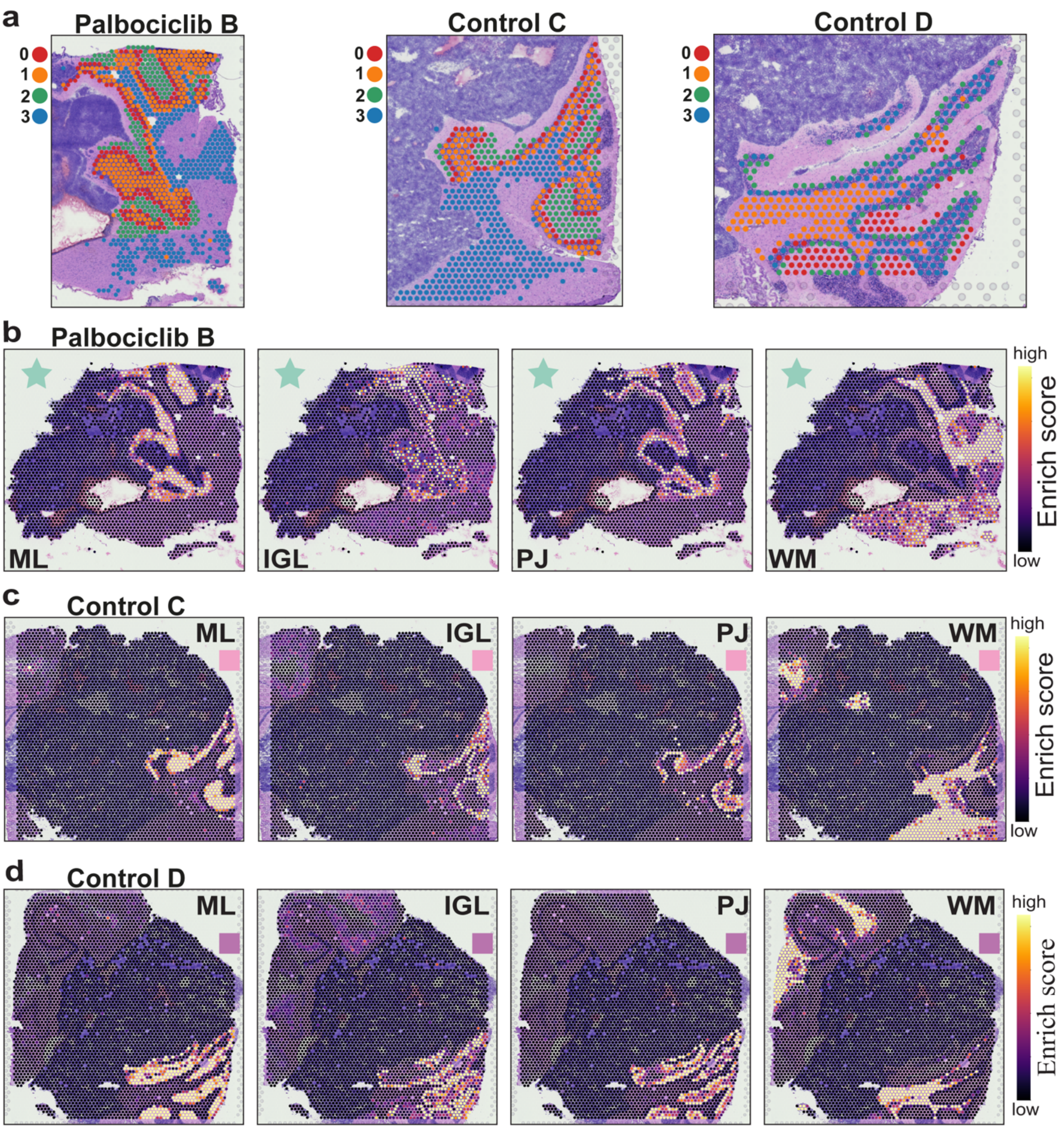

Supplementary Figure S2: Structures of mouse histology can be identified using unsupervised clustering across each Visium spatial transcriptomics samples, with high resolution and additional information about heterogeneity in gene expression even within one cluster.

a. Unsupervised clustering results for mouse tissue regions for samples Palbociclib B, Control C, and Control D, respectively; showing the clustering strongly corresponds to known cerebellum structures clear from the H&E. Colours and numbers indicate different clusters.

b-d. Per-spot enrichment results for top 10 marker genes of each cluster. Data for samples Palbociclib A for sample Palbociclib B, Control C, and Control D are shown. Regions of high enrichment correspond to the clustering results and well-defined cerebellum structures (ML: Molecular layer, PJ: Purkinje, IGL: internal granule layer, WM: White Matter layer).

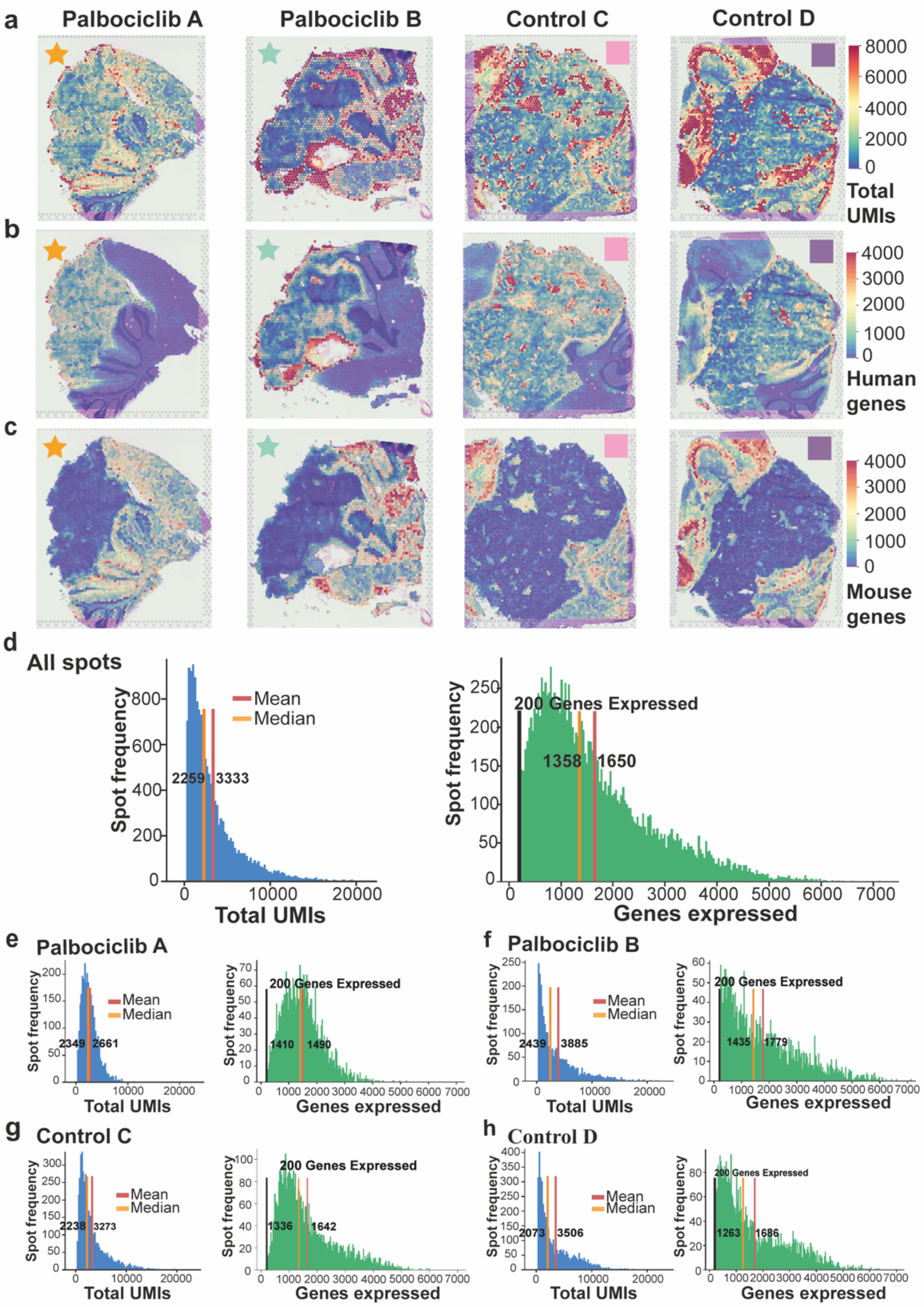

Supplementary Figure S3. Spatial visualisation and quality control assessment of gene expression within Medulloblastoma PDOX (MB-PDOX) mouse brains.

a. The total number of unique molecular identifiers (UMIs) per spot are visualised at their position within MB-PDX sections. The spots are coloured according to their detected UMI content (blue = low number of UMIs, yellow = medium number of UMIs, red = high number of UMIs).

b. Visium spatial RNA-seq detects human genes within the human tumour regions. The numbers of human genes detected for each spot across for each sample are shown (blue = low number of human genes, yellow = medium number of human genes, red = high number of human genes).

c. Equivalent to B, but for mouse genes only.

d. Histograms show the distribution of total UMIs detected for data from all spots across all samples (left, blue) and that for the number of genes expressed (right, green). The mean and median of each respective measurement are indicated in-text next to red and orange vertical lines, respectively. The black vertical line on the green histogram indicates a minimum of 200 genes expressed per spot was used to filter extremely low-quality spots.

e-h. The same as **d**, except for data is for each separate sample.

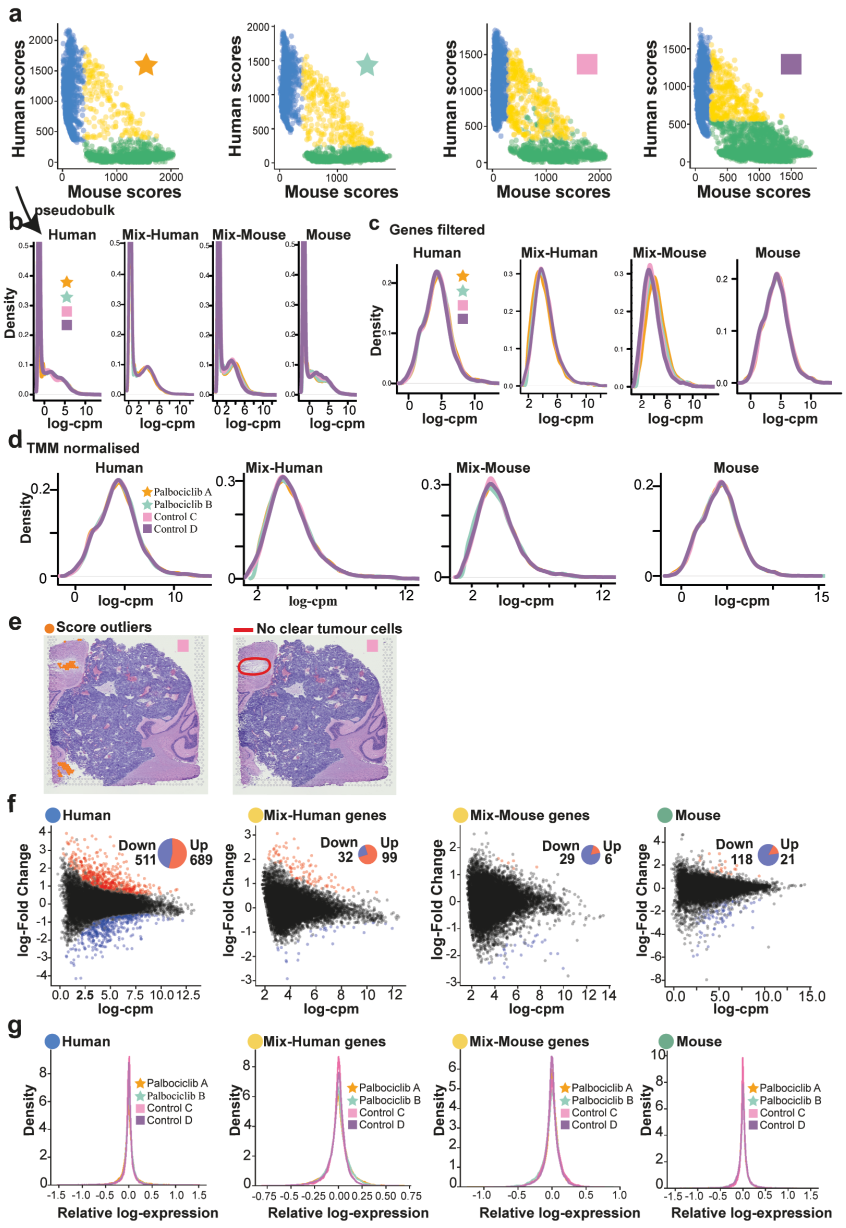

Supplementary Figure S4. Diagnostic plots to assess technical variation and normalisation for gene expression analysis of Visium ST-seq data.

a. Scatter plots with the sum of normalised human gene expression on the y-axis (normalised UMI count) and mouse gene expression on the x-axis per spot, allowing for classification of spots into species type. Spots are classified as human (green), mix (yellow, containing both human and mouse genes) or mouse (blue) based on whether they predominantly express one species' genes or a combination of both (see Methods).

b. The distributions of gene expression as raw log-cpm (log-counts-per-million) for each sample after pseudobulking (see Methods) for each of the human (left), mix (middle; including human genes in mix or mouse genes in mix, denoted as mix-human and mix-mouse respectively) and mouse (right) spots.

c. The same data as **b**, except that data were filtered to remove lowly expressed gene based on log-cpm cutoffs. The very high peaks for lowly expressed genes in B are not observed and the distributions become closer to Gaussian distributions.

d. The same data as **b**, except for log-cpm normalisation using trimmed-mean-squares (TMM) to estimate size factors after gene filtering. After filtering the data were more normally distributed. No sign of technical variation was observed among the four samples.

e. Adjustment of spot assignment based on pathological annotation. (Left) outlier spots in sample Control C which were relabelled as 'mouse' as opposed to 'mix' spots (see Methods). (Right) pathologist annotation indicating no clear evidence of tumours when examining the histology which motivated the relabelling of the outlier spots.

f. Scatter plots showing differentially expressed genes detected across the range of gene expression, suggesting that data were appropriately normalised with no bias towards highly or lowly abundant genes. Each point in the scatter plot is a gene, with red points indicating upregulated genes under Palbociclib treatment in MB and blue indicating downregulated genes. The y-axis is the log2-Fold Change from the Limma-Voom differential expression (DE) analysis comparing Palbociclib-treated against control samples. The x-axis is the log2-cpm average gene expression. The pie charts display the proportion of up- and down- regulated genes. From left-to-right, the described scatter plot is repeated for DE analyses comparing pseudobulked human spots/genes, mix spots with human genes, mix spots with mouse genes, and mouse spots/genes.

g. The density of the Relative Log Expression (RLE) values for each gene in each sample pseudobulked (from left-to-right) by human spots/genes, mix spots/human genes, mix spots/mouse genes, and mouse spots/genes. A distribution around 0 for each sample indicates the data were appropriately normalised for differential expression analysis with no systematic shift in the relative gene expression between the pseudobulked samples in each comparison.

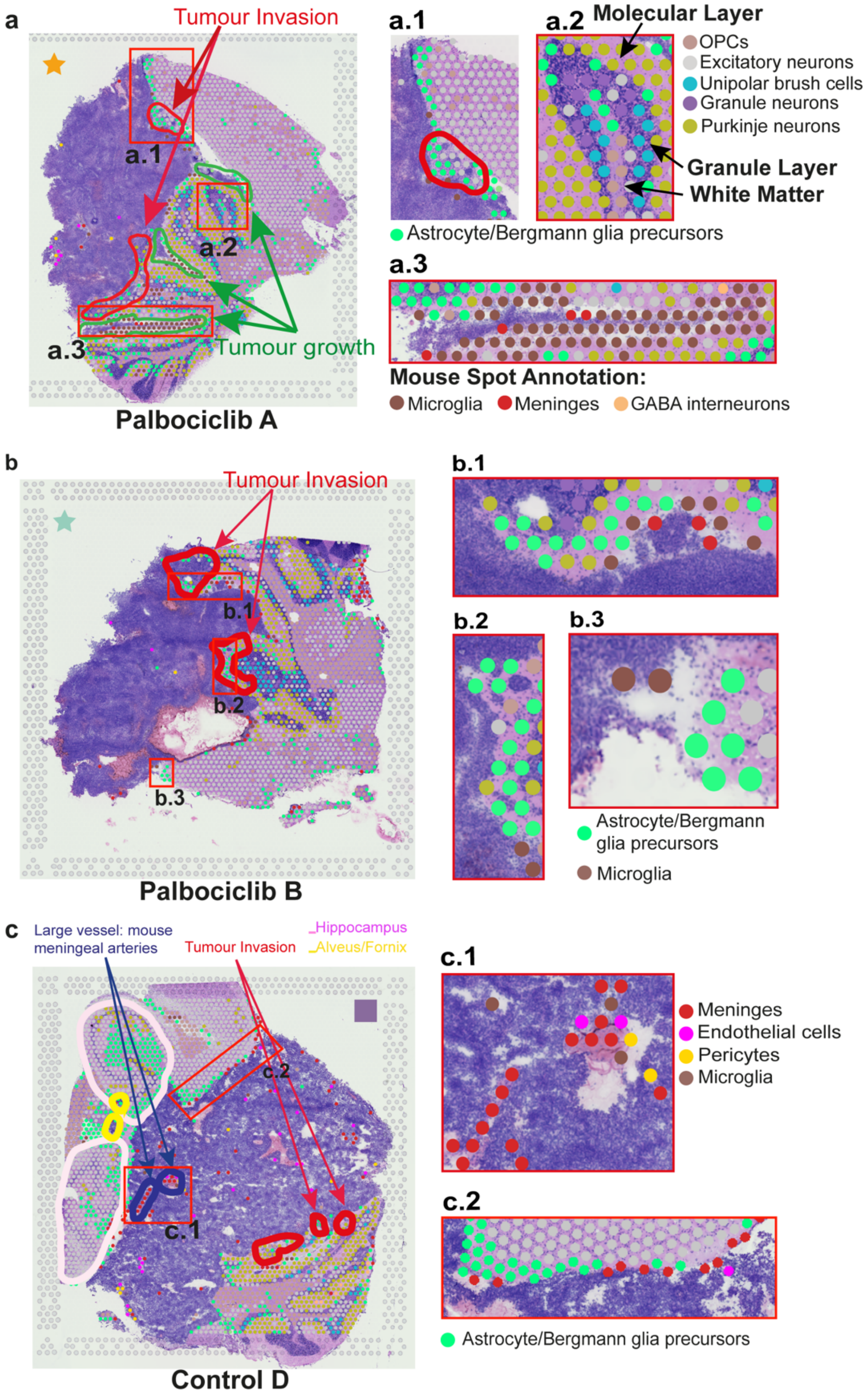

Supplementary Figure S5. Data-driven cell type detection for spatial transcriptomics spots highly correlated with pathologist's annotation based on mouse histology and had a high resolution sufficiently reflecting cell-type heterogeneity within small tissue regions.

a. Independent pathology annotation (shown as contours/circles) of sample Palbociclib A overlaid on top of Visium spots cell types (shown as different colours). **a1-a3**. Enlarged regions in Palbociclib A (red boxes). **a1**. Enlarged region showing astrocytes at the tumour/mouse interface, and a tumour invasion region annotated by the pathologist in red. **a2**. Enlarged region showing the different layers of the mouse cerebellum and the spot cell types; indicating the expected cell types in their respective cerebellum layers. Purkinje cells were primarily in the molecular layer; granule, unipolar brush, and excitatory neurons were dominant in the granule layer; while the white matter was predominantly oligodendrocyte precursor cells (OPCs). **a3**. Enlarged region annotated by the pathologist as 'tumour growth'; the dominant mouse cell types interfacing with the tumour are microglia, astrocyte/Bergmann glia, and meninges.

b. Independent pathology annotation of sample Palbociclib B overlaid on top of Visium spots cell types. **b1-b3**. Enlarged region in Palbociclib B (red boxes). **b1-b2**. Enlarged interface region from **b** (red boxes) which overlaps pathologist annotation 'Tumour invasion' indicates astrocytes, microglia, and meninges as dominant cell types. **b3**. Enlarged interface region indicates astrocytes and microglia as the dominant cell types in the interface spots.

c. The same as **a, b** for sample Control D. **c1-c2**. Enlarged region in Control D (red boxes). **c1**. Enlarged region annotated by the pathologist as 'Large vessel: mouse meningeal arteries'. Meninges, endothelial cells, pericytes, and microglia are the predominant cell types in these regions. The dominant cell type for these areas are primarily blood-vessel related, in concordance with the pathologist annotation. **c2**. Enlarged region from sample Control D showing the tumour-mouse interface. astrocytes, meninges, and endothelial cells as the dominant cell types at the interface between the mouse and tumour tissue.

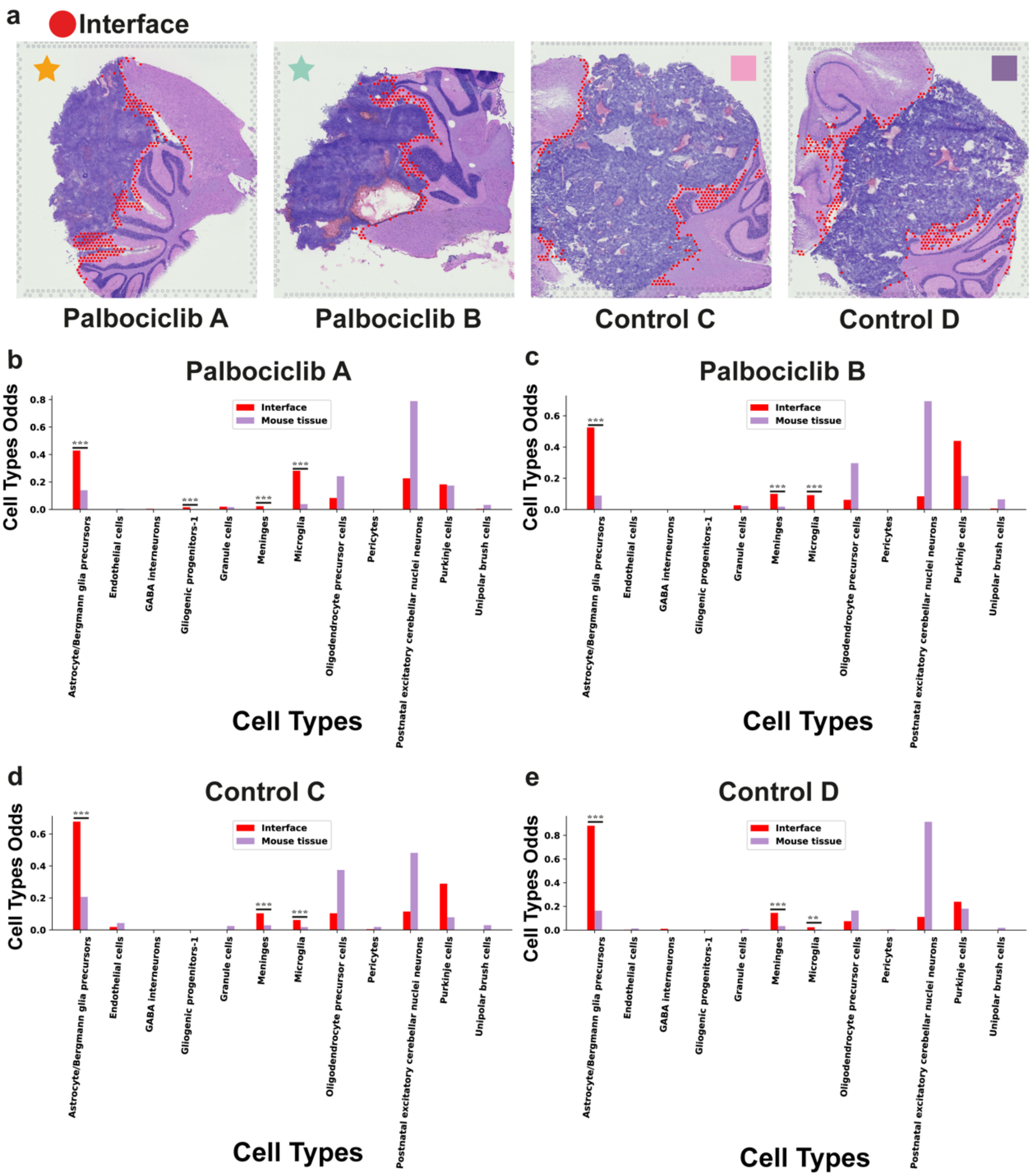

Supplementary Figure S6. Definition of spots at the tumour-mouse interface.

a. Spots at the tumour-mouse interface which were used to test for enrichment of astrocytes for samples (left-to-right) Palbociclib A, Palbociclib B, Control C, and Control D, respectively.

b-e. Gene expression of each cell types between interface and “mouse tissue” for samples Palbociclib A, Palbociclib B, Control C, and Control D, respectively.

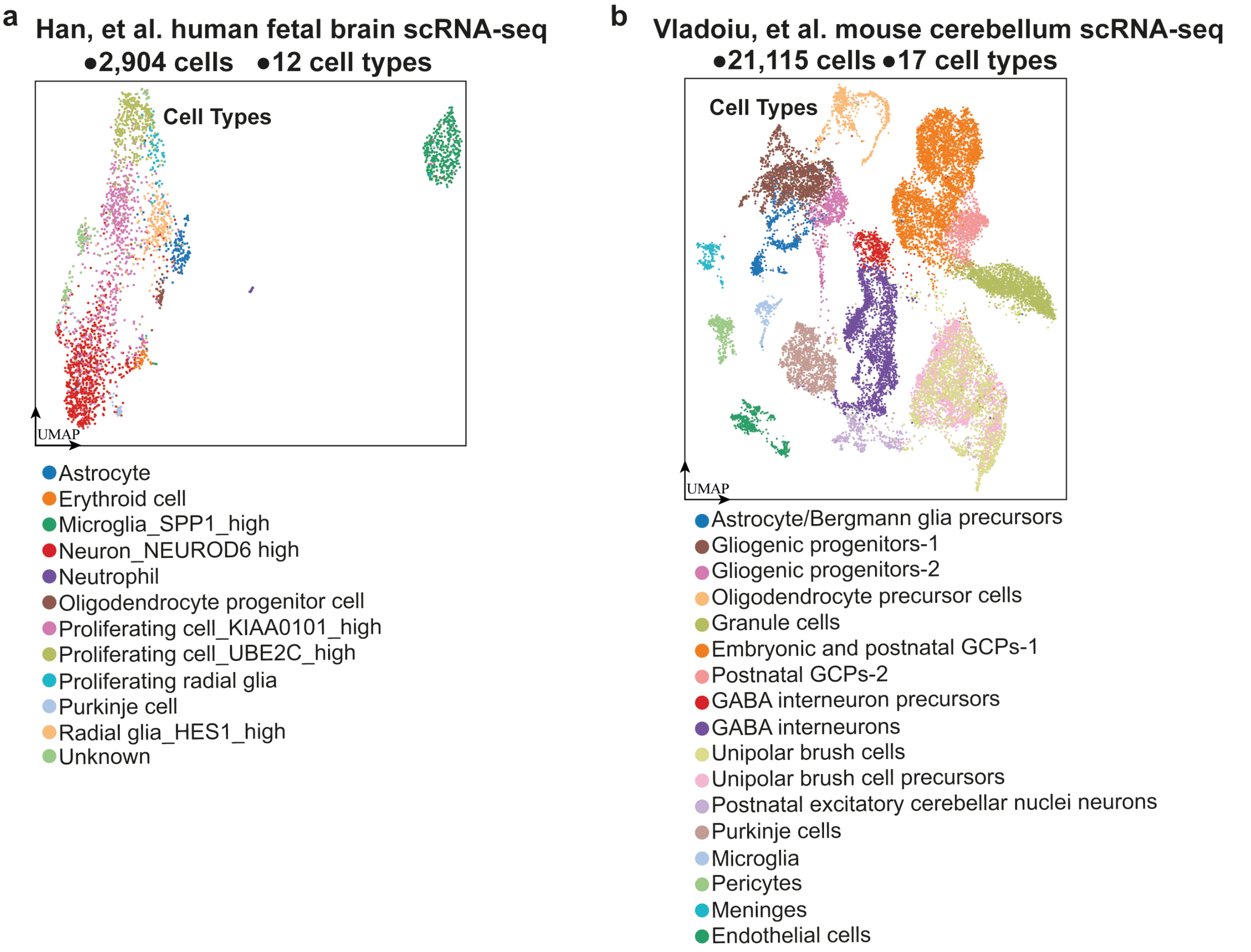

Supplementary Figure S7. Overview of the reference single cell RNA-seq data used for automated spot cell type identification.

a. The reference scRNA-seq data used to annotate human/mix spots (only human genes were used for the mix spots). The data was obtained from Han, et al. (2020), consisting of 2,904 cells spanning 12 different cell types from 13 week male human foetal brain.

b. The reference scRNA-seq data used to annotate mouse/mix spots using mouse genes. The data was obtained from Vladoiu, et al. (2019), consisting of 21,115 cells spanning 17 cell types once subsetted to cell types only present at postnatal day 7 (P7).

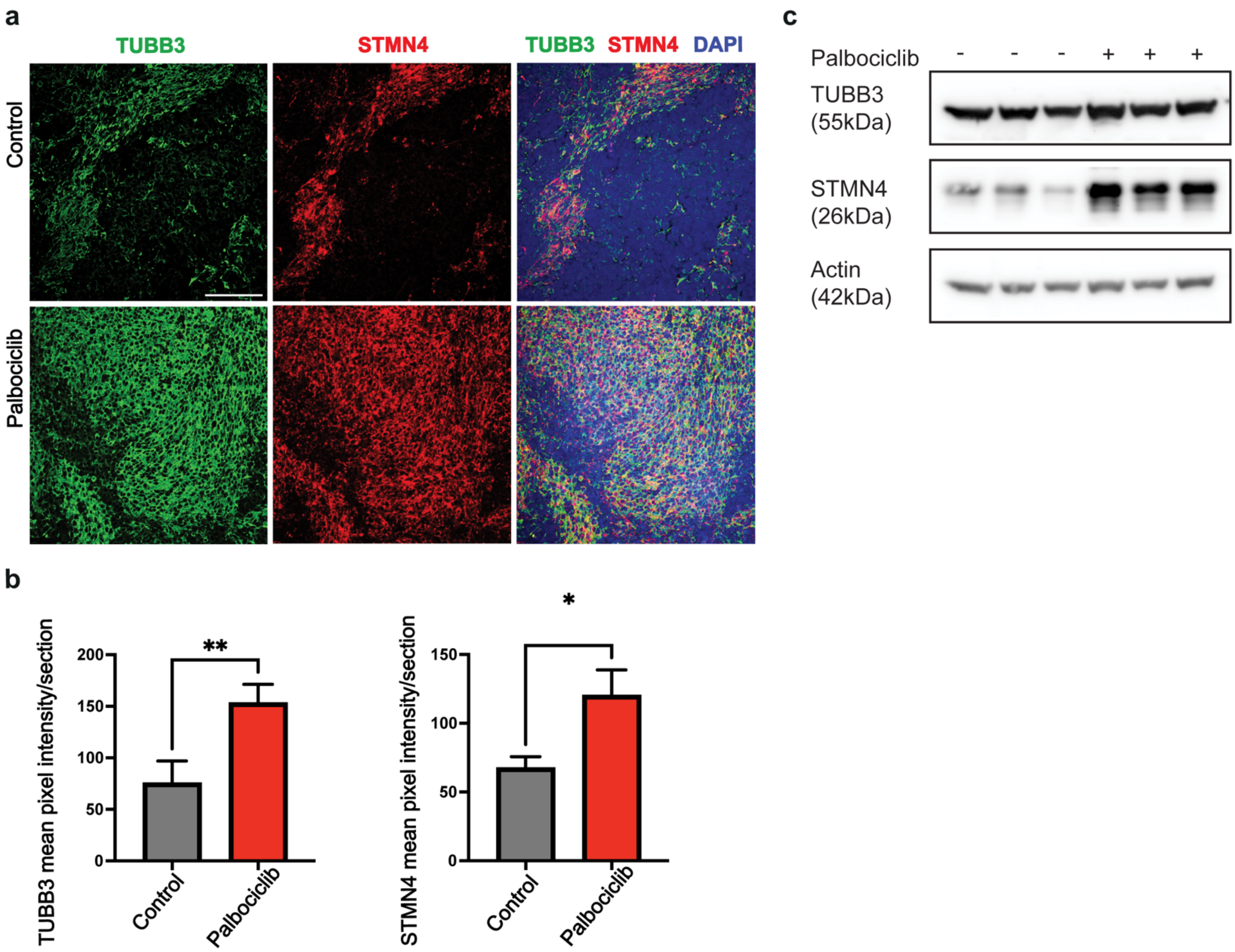

Supplementary Figure S8. Palbociclib-treatment induces the expression of genes associated with neuron differentiation in SHH MB.

a.Representative images of two markers of neural differentiation (TUBB3 and STMN4) in immunofluorescence-labelled Med-1712FH orthotopic tumours following Palbociclib treatment (**a**, top panel) compared to untreated control tumours (**a**, bottom panel). Sections were counterstained with DAPI. Scale bar 100 µm.

b. Quantitative analysis of TUBB3 (**b**, left) and STMN4 (**b**, right) staining in Med-1712FH tumours. Mean pixel intensity was quantified for each marker for vehicle (n = 3) and drug-treated tumours (n = 3) using ImageJ software. Data are presented as the mean ± SEM. Statistical evaluation was performed using an unpaired two-tailed t-test ($\alpha = 0.05$) with Welch's correction. Statistically significant differences are indicated (*$p \leq 0.05$; **$p \leq 0.01$).

c. Immunoblot of protein extracts from untreated (n = 3) and Palbociclib-treated (n = 3) Med-1712FH orthotopic tumours for expression of TUBB3, STMN4 and Actin. Immunoblots were imaged using the BioRad ChemiDoc MP Imager.

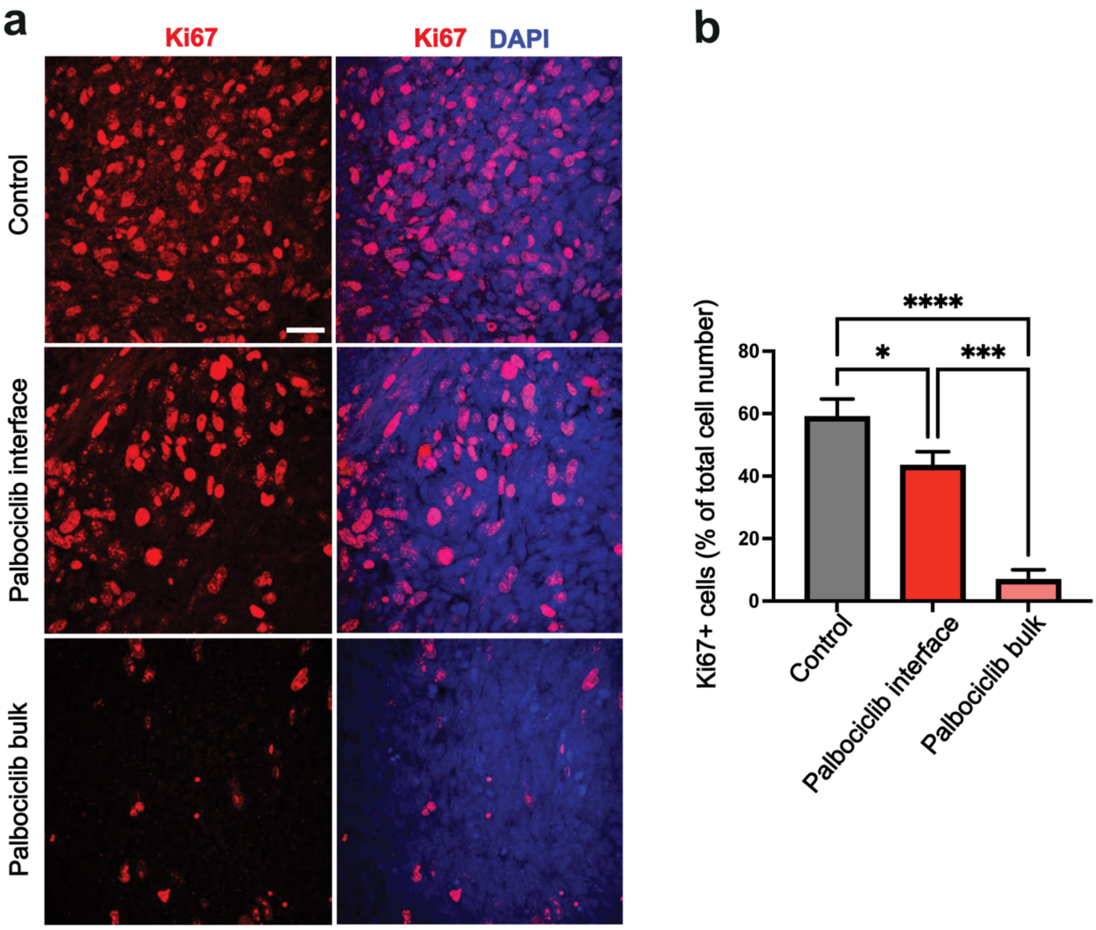

Supplementary Figure S9. Spatial response of cell proliferation to Palbociclib treatment in SHH MB.
a. Representative images of cell proliferation (Ki67) in immunofluorescence-labelled Med-1712FH orthotopic tumours in untreated control tumours (**a**, top panel) compared to the interface (**a**, middle panel) and bulk (**a**, bottom panel) regions of tumours following Palbociclib treatment. Sections were counterstained with DAPI to determine total cell number. Scale bar 20 μm.
b. Quantitative analysis of tumour cells staining positive for Ki67 in untreated control tumours (n=3) compared to the interface and bulk tumour regions of Palbociclib-treated (n=3) tumours. Data are presented as the mean ± SEM. Statistical evaluation was performed using a multiple t-test with Tukey's correction. Statistically significant differences are indicated (*p ≤ 0.05; ***p ≤ 0.001; ****p ≤ 0.0001).

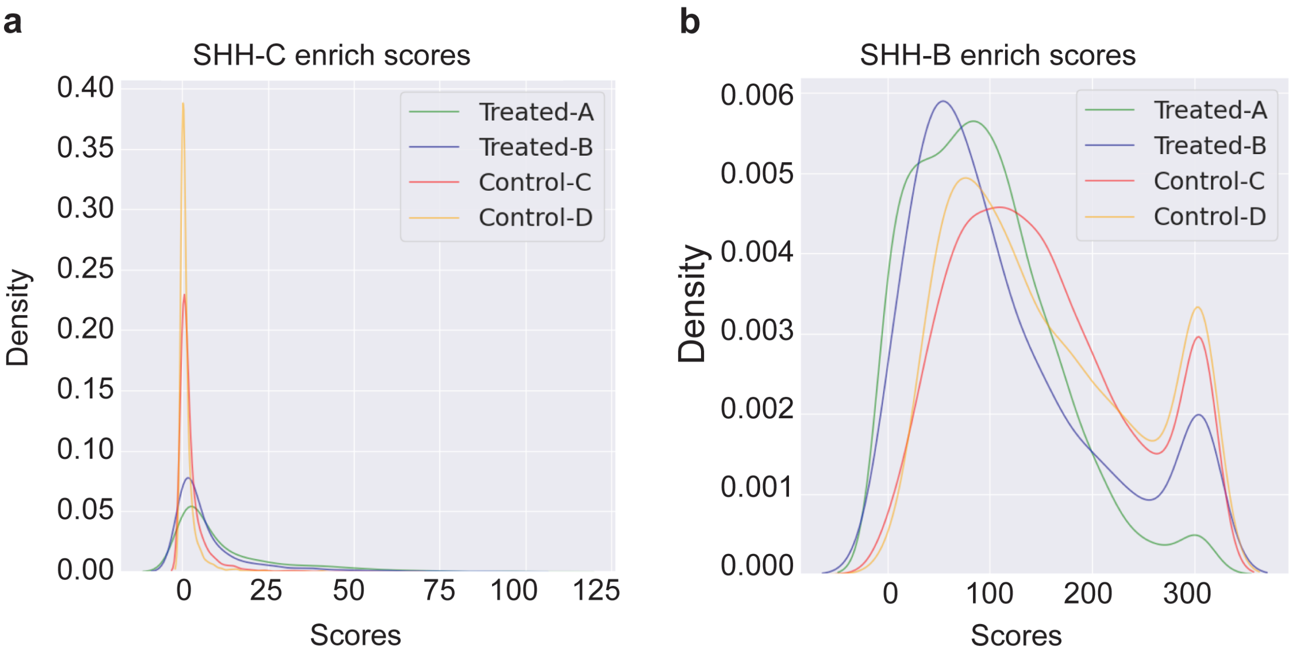

Supplementary Figure S10. Gene set activity scores of SHH-C (**a**) and SHH-B (**b**) groups. Density plots show normalised area, where each plot has a total density of one, allowing us to compare between plots, clearly showing the two drug-treated PDOX have higher SHH-C activities and lower SHH-B activities.

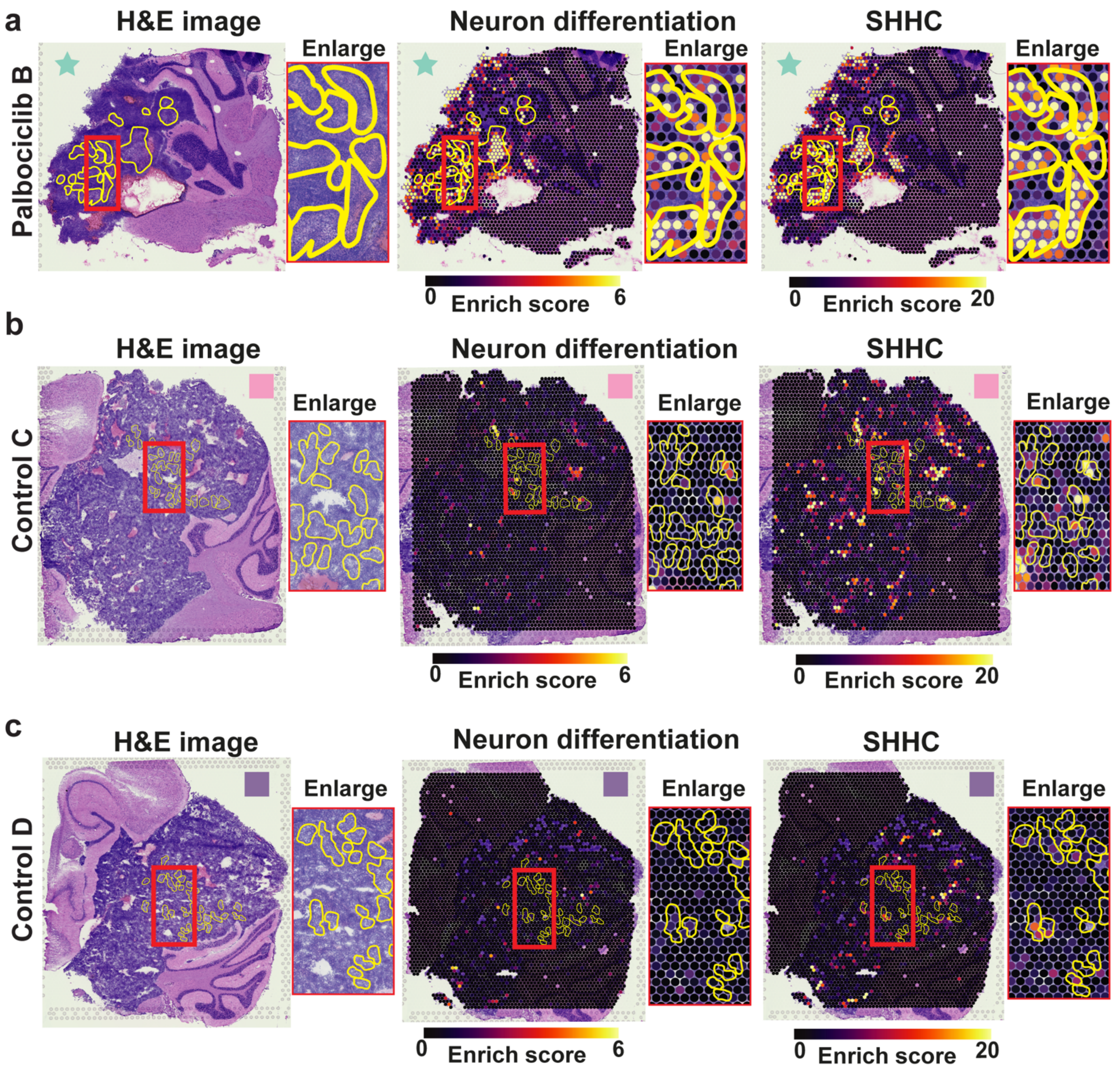

Supplementary Figure S11. Per-spot enrichment analysis of DE neuron differentiation genes and neuronally differentiated SHH-C subpopulation corroborates pathologist “pale island” annotations.

a. Comparision between pathologist annotations from H&E images (left) and per-spot enrichment scores of differentially expressed (DE) neuron differentiation genes and SHH-C in Palbociclib A. ‘Pale island’ annotations are indicated in yellow, with enlarged sections in red boxes.

b-c. Equivalent to A except for samples Control C and Control D.

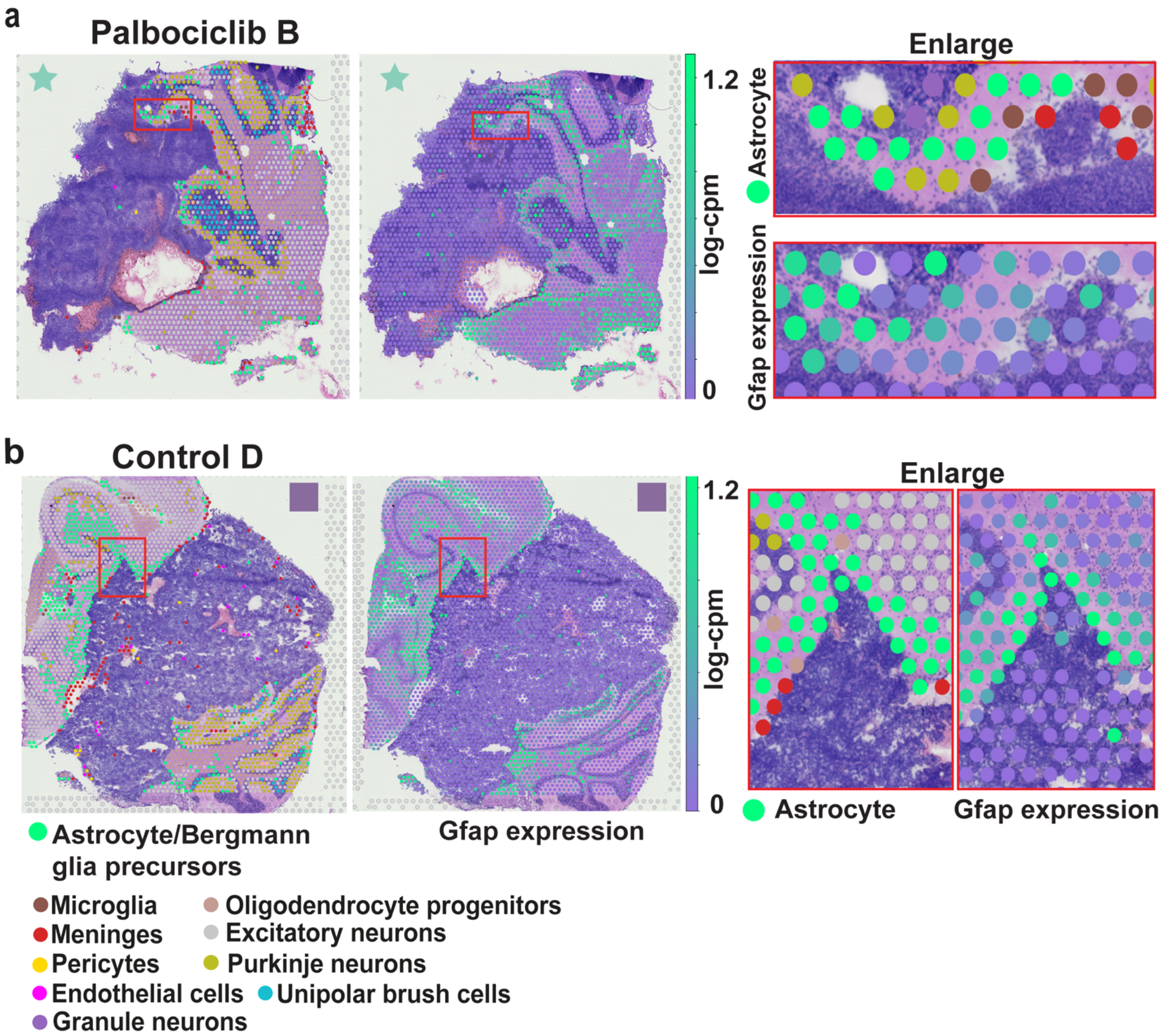

Supplementary Figure S12. Analyses of mouse-only tissue region shows that mouse astrocytes localise to the tumour-mouse interface.
a. Sample Palbociclib A with overlaid mouse spot annotations (left) and Gfap gene expression (right, in log-cpm (log-counts-per-million)). The red box indicates the region of interest enlarged to the far right. Cell types are displayed as different colours. Overall, there is concordance between Gfap gene expression in spots and astrocyte cell types; with both localised to the interface region.
b. Equivalent to **a**, except depicting sample Control D.

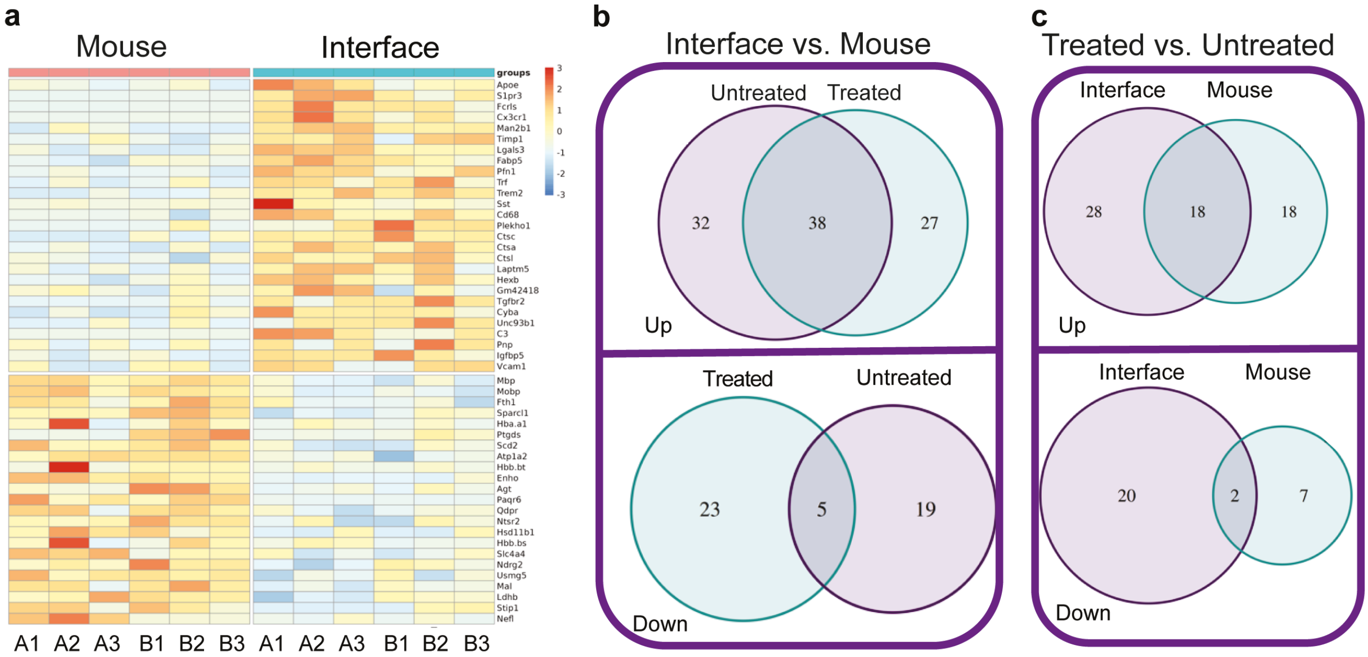

Supplementary Figure S13. Differential expression analysis of astrocytes at the interface.

a. heatmap showing the expression of genes that are uniquely upregulated (red) or downregulated (blue) in astrocyte spots within the mixed tumour-microenvironment interface compared to those annotated as mouse only. The X-axis shows pseudobulk replicates of the biological replicates for drug-treated PDOX samples, Palbociclib A and Palbociclib B. Each sample has three pseudopools of replicates (A1, A2, A3 for Palbociclib A and B1, B2, B3 for Palbociclib B).

b. DE genes between the spots annotated as astrocytes in the mixed tumour-microenvironment interface compared to those annotated as mouse only within the same tissue. Shared or unique between the drug-treated and untreated PDOX are shown. The Venn diagram indicates upregulated genes (top) and downregulated genes (bottom).

c. DE genes between the spots annotated as astrocytes in the mixed tumour-microenvironment interface compared to those annotated as mouse only between the treated and untreated samples. Shared or unique between the drug-treated and untreated PDOX are shown. The Venn diagram indicates upregulated genes (top) and downregulated genes (bottom).

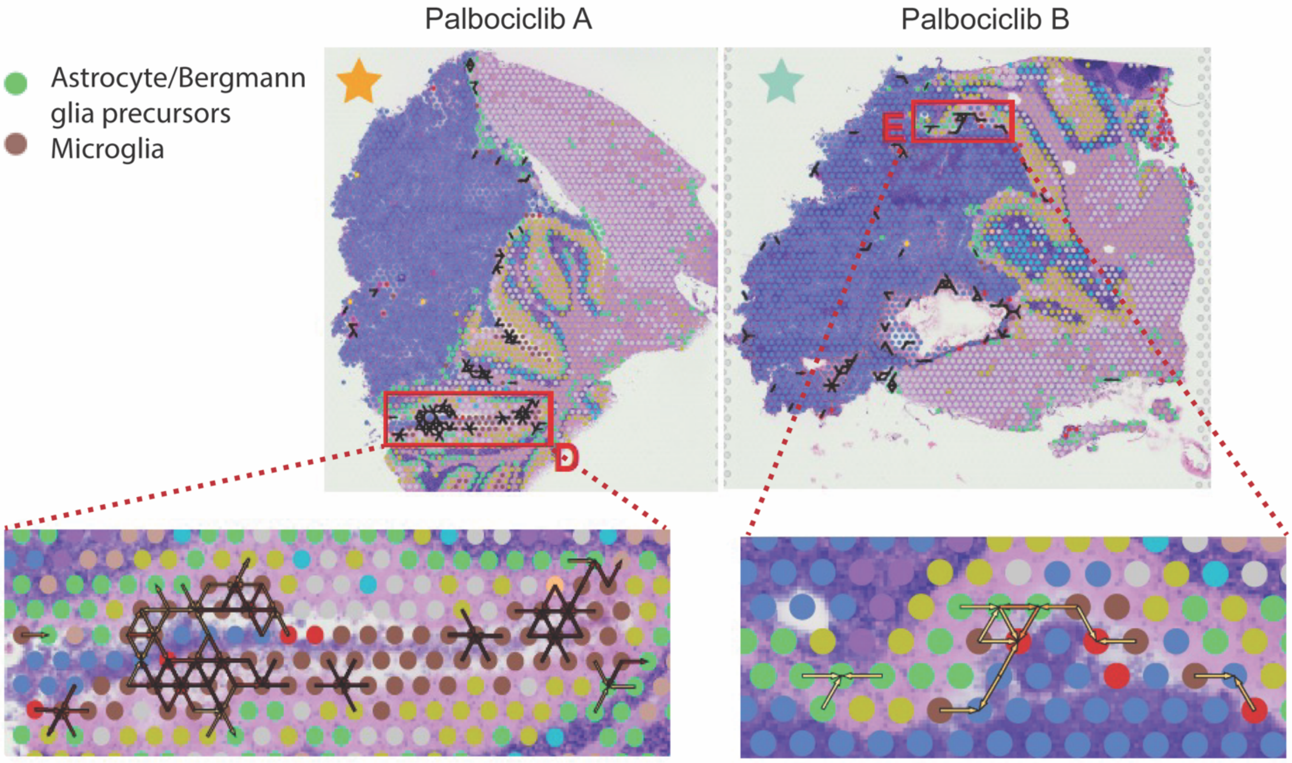

Supplementary Figure S14. Spatial interaction analysis. Astrocyte and microglia interaction is enriched specifically at the mixed tumour-microenvironment interface. Both Palbociclib A and B are shown.

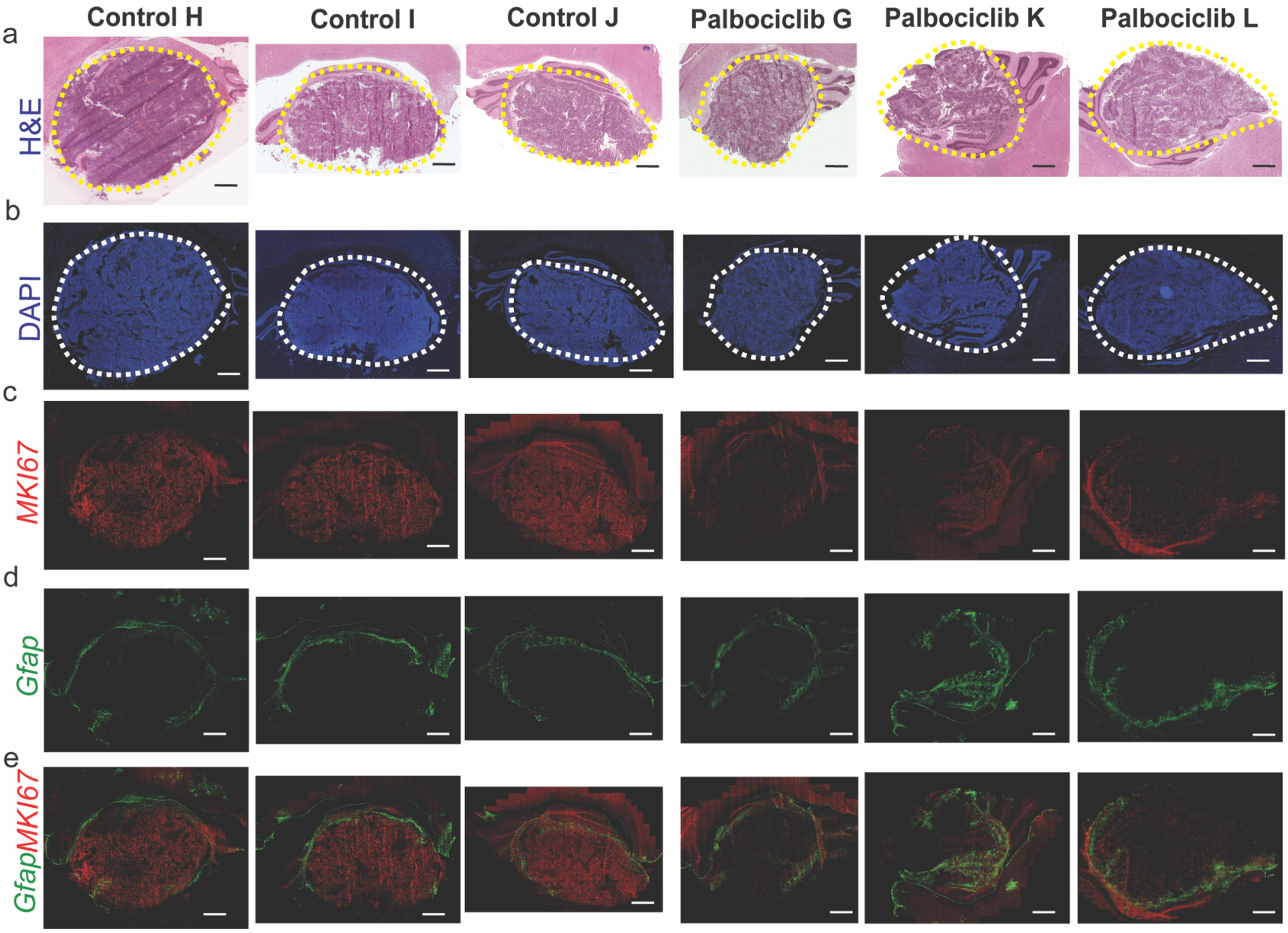

Supplementary Figure S15. The expression of cell proliferation marker *MKI67* is localised tumour regions with expression of astrocyte marker, *Gfap*. Scale bar 1mm.

a. High resolution H&E images of drug-treated and untreated PDOX with tumour volumes outlined in yellow dashed lines.

b. DAPI staining of control and drug-treated PDOX with tumour volumes outlined in white dashed lines.

c. Target RNA molecule expression of cell proliferation marker *MKI67* using RNAscope in control and drug-treated PDOX.

d. Target RNA molecule expression of astrocyte marker *Gfap* localised to the tumour-mouse interface region. (e) Merged image of *MKI67* expression with *Gfap* expression across drug-treated and untreated PDOX.

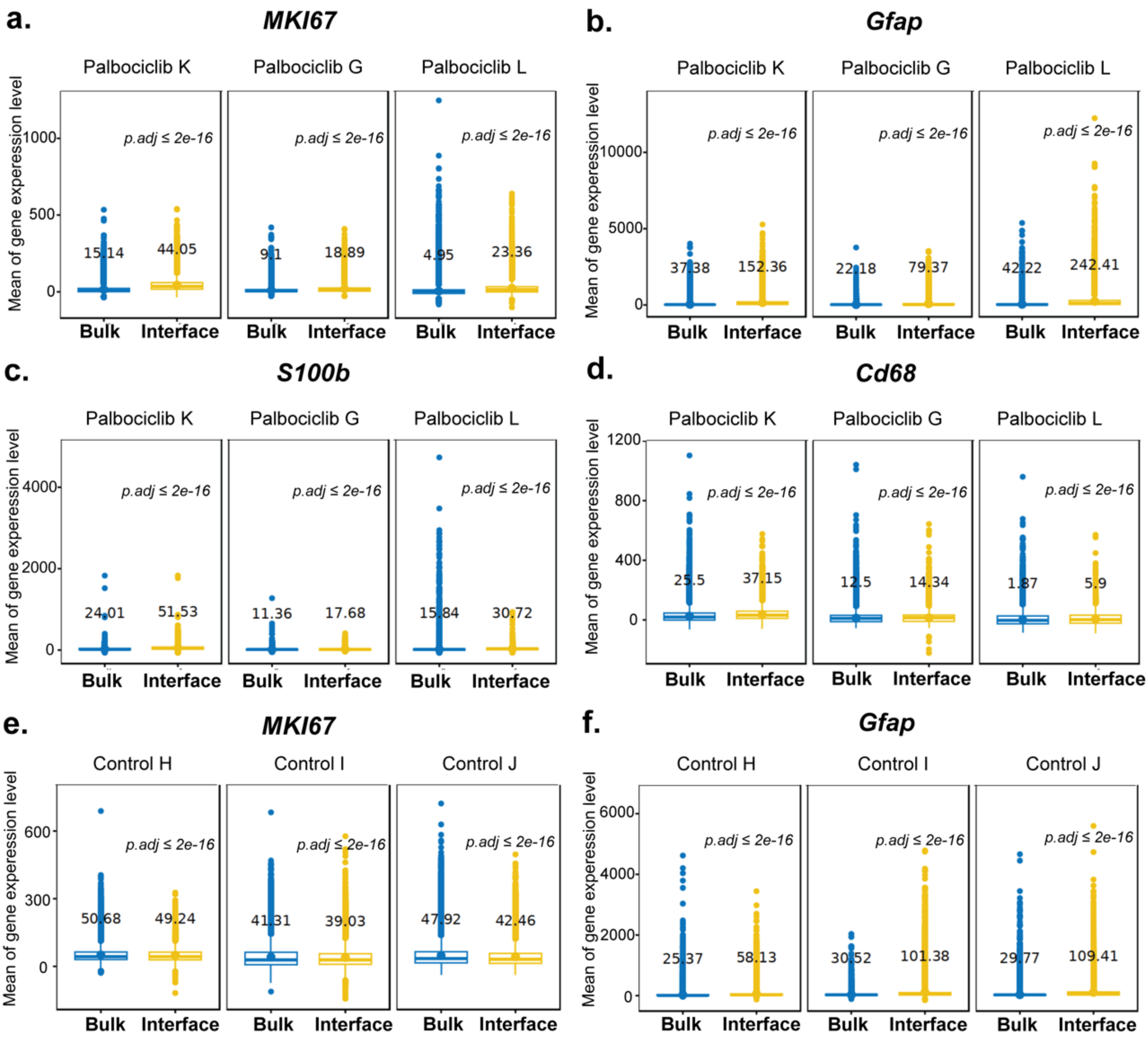

Supplementary Figure S16. Quantitation of gene expression levels as measured by smRNA-FISH/RNAscope for tumor cell proliferation marker MKI67 (a), astrocyte markers Gfap (b) and S100b (c), and microglial marker Cd68 (d) between the interface and bulk regions of Palbociclib-treated PDOX. Quantification of MKI67 (e) and astrocyte marker Gfap (f) was also performed to compare the interface and bulk regions of untreated PDOX. Statistical evaluation was performed using a Mann-Whitney test with Bonferroni correction. The numbers of measured cells for each of the three treated samples are Palbociclib K (Bulk: 101,048 cells; Interface:58,180 cells), Palbociclib G (Bulk: 127,850 cells; Interface: 49,211 cells), Palbociclib L (Bulk: 97,977 cells; Interface: 36,389 cells). Control H (Bulk: 163,836 cells; Interface: 34,929 cells), Control I (Bulk: 120,736 cells; Interface: 49,719 cells), Control J (Bulk: 164,878cells; Interface: 53,924 cells).

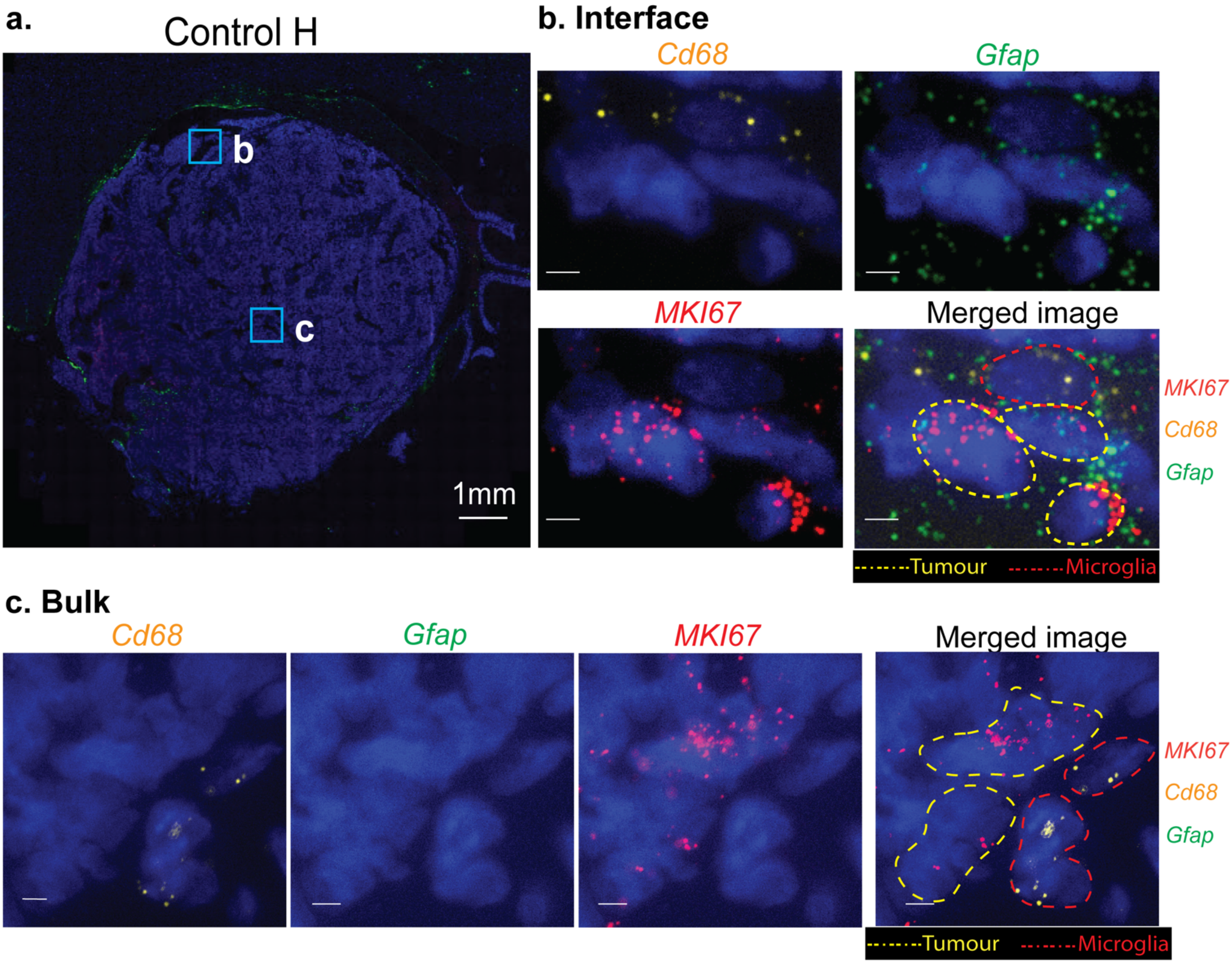

Supplementary Figure S17. RNAScope analysis was used to investigate cell types comprising a multi-lateral network associated with tumour progression in untreated PDOX. (a) DAPI image overview of a untreated PDOX, Control H (b) Representative target RNA molecule expression at a single cell level using RNAscope for gene markers of cell types and associated ligands and receptors of proposed hypothesis microglia (Cd68), astrocytes (Gfap) and proliferating tumour cells (MKI67) within the tumour microenvironment interface and (c) tumour bulk. Scale bar: 5µm.
